# Characterizing living ocular bacterial communities and the effects of antibiotic perturbation in house finches

**DOI:** 10.1002/mbo3.1398

**Published:** 2024-02-04

**Authors:** Chava L. Weitzman, Dana M. Hawley, Bahman Rostama, Meghan May, Lisa K. Belden

**Affiliations:** ^1^ Department of Biological Sciences Virginia Tech Blacksburg Virginia USA; ^2^ Research Institute for the Environment and Livelihoods Charles Darwin University Darwin Northern Territory Australia; ^3^ Department of Biomedical Sciences University of New England Biddeford Maine USA; ^4^ Division of Livestock, Poultry, and Dairy Idexx Laboratories Westbrook Maine USA

**Keywords:** antibiotic disruption, cefazolin, conjunctival microbiome, *Haemorhous mexicanus*, microbial ecology, ocular bacteria

## Abstract

DNA‐based methods to measure the abundance and relative abundance of bacterial taxa can be skewed by the presence of dead or transient bacteria. Consequently, the active, functional members of the community may be a small subset of the detected bacterial community. This mismatch can make inferences about the roles of communities in host health difficult and can be particularly problematic for low‐abundance microbiomes, such as those on conjunctival surfaces. In this study, we manipulated bacterial communities on bird conjunctiva with a bacteriostatic antibiotic, reducing bacterial activity while preserving viability, to identify the living and active conjunctival communities using comparisons of 16S ribosomal DNA and RNA in paired samples. DNA amplicons included many more sequence variants than RNA amplicons from the same communities, with consequent differences in diversity. While we found that changes in communities in DNA samples broadly represent shifts in the living (RNA‐amplicon) communities, assessments of community function may be better described by RNA samples, reducing background noise from dead cells. We further used these data to test RNA:DNA ratios, used in other microbiological contexts, to detect shifts in bacterial activity after antibiotic disruption but were unable to detect changes in bacterial activity with this method.

## Graphical abstract

Sequencing DNA versus RNA samples can overestimate bacterial community members from the eye of a small songbird but still provide an adequate representation of bacterial communities and changes therein. Photo credit: Sara R. Teemer.

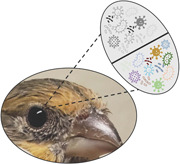

## 1 INTRODUCTION

Our understanding of the structure and function of bacterial communities across environments has been greatly expanded with the increased ease of high‐throughput sequencing technologies (Bharti & Grimm, 2021). Bacterial communities are now commonly described using 16S rRNA gene amplicon sequencing, leading to important advances in our understanding of microbial community structure in both environmental and host‐associated systems. For example, in multiple animal host systems, amplicon sequencing has highlighted that infection and disease are associated with altered host‐associated bacterial community structure (Allender et al., 2018; DeCandia et al., 2020; Jani & Briggs, 2014; Thomason, Leon, et al., 2017). However, while 16S rRNA gene amplicon sequencing has proven extremely useful in detecting meaningful differences between bacterial communities, it cannot differentiate between living and dead cells in a community. Thus, additional data to characterize the living or active bacterial members of host‐associated microbiomes may be useful in detecting the most important taxa in a community.

Among environmental and host‐associated systems, a large proportion of the DNA community may represent dead or inactive members, often depending on nutrient availability or the presence of bacteriostatic compounds (Bowsher et al., 2019; Jones & Lennon, 2010; Lennon et al., 2018). Bacterial microbiomes with low biomass pose an additional hurdle to understand, as amplicon data may be disproportionately affected by DNA from nonparticipating cells. In low biomass systems, focusing on the living, active members with RNA‐based amplicons should provide a better representation of the communities of interest. RNA can be used to better represent the biologically active portion of communities because RNA degrades faster than DNA (e.g., Emerson et al., 2017; Li et al., 2017; Liu & Yang, 2020). However, few ecological studies of host‐associated microbiomes using amplicon sequencing incorporate data from cDNA reverse‐transcribed from 16S rRNA (hereafter RNA; but see, e.g., Caughman et al., 2021; Ofek et al., 2014).

Beyond describing living bacteria, incorporating data from RNA with that from DNA (from 16S rRNA gene) through ratios of RNA:DNA copies for each bacterial taxon may provide an estimate of bacterial activity in samples. This method is applied to discern more or less active bacteria, but can also be used to detect bacteria that have entered a reversible state of dormancy or extremely slowed growth, a strategy some bacteria use when starved or faced with other extreme conditions (Gray et al., 2019; Jones & Lennon, 2010; Rittershaus et al., 2013). RNA:DNA ratios have been employed most extensively to describe communities in soil systems (Bowsher et al., 2019; Sorensen & Shade, 2020), where bacterial dormancy is quite common and often associated with carbon fluxes, but has also been investigated in other extreme environments, such as hypersaline lakes (Aanderud et al., 2016). In contrast, this method is not typically applied to host‐associated microbiomes. Importantly, not everyone agrees about the utility of RNA amplicons and RNA:DNA ratios; their use has been challenged based on extracellular DNA and the uncoupling of rRNA production with activity, growth, and dormancy (Blazewicz et al., 2013; Dlott et al., 2015).

In this study, we used paired samples of bacterial 16S DNA and RNA from house finch (*Haemorhous mexicanus*) ocular swabs to assess differences between the community that is commonly described (i.e., that from the DNA sample) and the living members of the bacterial community (the RNA sample). Despite their low biomass, ocular bacteria interact with their hosts in important ways and can alter disease outcomes (Ozkan et al., 2017; St. Leger & Caspi, 2018). In house finches, ocular bacterial microbiomes can protect their hosts from bacterial conjunctivitis infection, but this outcome depends on microbiome composition (Thomason, Mullen, et al., 2017; Weitzman et al., 2021). In light of the apparent importance of ocular community composition in protection from conjunctival disease, we chose a subset of samples from a previous experiment (Weitzman et al., 2021) to gain a better understanding of the representation of the living and active members of the community.

Just as dormancy dynamics can be experimentally manipulated in soil systems through nutrient deprivation, we can use antibiotics to manipulate communities in hosts. In Weitzman et al. (2021), we used topical administration of the bacteriostatic β‐lactam antibiotic cefazolin to push some members of the ocular community into an inactive state, with a measured reduction in replicating bacteria in birds given the antibiotic, detected using culture‐based methods. While culture‐based methods are particularly useful in quantifying microbial viability, not all bacteria in microbiomes are readily culturable under standard conditions (Walke et al., 2015). Here, we use DNA‐ and RNA‐based amplicon sequencing to better understand the living and active component of the ocular microbiome after antibiotic exposure, given that DNA approaches alone may not adequately capture the action of a bacteriostatic drug. By preventing cellular growth, bacteriostatic antibiotics may also be used to test the utility of RNA:DNA ratios for describing active and inactive bacteria in hosts.

We use the whole RNA and DNA datasets to identify the living portion of the total community, but then focus this study on the more abundant members of the bacterial communities that are more likely to be functionally important for the host. After describing the abundant, active members of the ocular communities, we assessed shifts in the DNA portion of the bacterial community, “living” community (defined here as members of the DNA community also present in the RNA community), and “active” community members (which we quantify here with RNA:DNA ratios) after antibiotic community disruption. We used these data to test the hypotheses that (1) RNA samples will be less diverse than their DNA counterparts as a consequence of the method used to define the living community (see Section 2), (2) the detected bacterial community composition will shift due to the bacteriostatic antibiotic, reflected in RNA but not DNA samples, and (3) some bacteria, particularly those previously found to be susceptible to cefazolin, will experience a shift toward inactivity (lower RNA:DNA ratios) due to bacteriostatic antibiotic treatment.

## 2 METHODS

Hatch‐year house finches were captured in July 2019 in Montgomery County and Radford, Virginia for a separate experiment on the effects of perturbing the resident ocular microbiome on disease caused by the conjunctival pathogen *Mycoplasma gallisepticum* (Weitzman et al., 2021). Birds were captured under VDGIF (061440) and USFWS (MB158404‐0) permits. Only birds with no signs of mycoplasmal conjunctivitis following capture were included in the study. Experimental birds were randomly divided between antibiotic and no‐antibiotic (control) treatments. Birds were single‐housed 6 days before receiving topical ocular antibiotics, provided a constant 12:12 photoperiod, and given access to food and water ad libitum.

We administered the β‐lactam antibiotic cefazolin diluted to 33 mg/mL in artificial tears (Bausch + Lomb Advanced Eye Relief Dry Eye) to perturb the resident microbiome, as previously described (Thomason, Mullen, et al., 2017; Weitzman et al., 2021). Antibiotics were given for 5 days, three times per day, by droplet instillation of 15 µL onto each eye. Control birds were caught three times per day at the same time with no further sham treatment to avoid altering the ocular microbes. We used culturing of ocular swabs to verify general antibiotic efficacy on the communities; results indicated a significant decrease in ocular bacterial growth among the experimental birds relative to controls (Weitzman et al., 2021).

We collected ocular microbiome samples using flocked swabs (Copan FLOQSwabs, Copan Diagnostics Inc.) lubricated with artificial tears to swab birds' conjunctiva, preserving the two swabs per bird into a single prepared tube of 300 µL Zymo DNA/RNA Shield (Zymo Research). We assessed the ocular microbiomes of each bird the day they were single‐housed (pretreatment) and immediately before the final antibiotic dose.

### 2.1 Ocular microbiomes

For the present study, we collected 14 samples from seven birds (pretreatment *n* = 7, postcontrol *n* = 3 [two male, one female], postantibiotic *n* = 4 [two males, two females]). We extracted DNA and RNA with the Zymo Quick DNA/RNA Microprep extraction kit (Zymo Research), eluting each in 15 µL DNase/RNase‐free water. RNA samples were converted to cDNA with reverse‐transcription PCR using Applied Biosystems High‐Capacity cDNA Reverse Transcription Kit (Applied Biosystems). We prepared samples for amplicon sequencing as previously described, amplifying a portion of the V4 region of 16S bacterial rRNA using 515F and barcoded 806R primers (Caporaso et al., 2012; Thomason, Leon, et al., 2017). Though we did not sequence environmental or extraction controls, we performed library prep on DNA and RNA from two environmental controls and one extraction control to verify that the input of environmental contamination in our samples was low. From Qubit quantitation, library‐prepped DNA and RNA samples from birds had higher values than the extraction and environmental controls (mean ± SD ng/μL, sample DNA: 16.3 ± 7.5; sample RNA: 31.9 ± 6.7; DNA controls: 2.4 ± 0.4; RNA controls: 7.5 ± 2.4). Amplicon sequencing was completed on an Illumina MiSeq using a 250 bp single‐end strategy at Harvard's Dana Farber Cancer Institute Molecular Biology Core Facilities, with samples from other experiments sharing the run.

Single‐end 251 bp sequence reads were demultiplexed using QIIME2 (Bolyen et al., 2019). With the DADA2 package in RStudio (Callahan et al., 2016; RStudio Team, 2020), we used the filterAndTrim function with otherwise default parameters to discard reads with more than two expected errors. Amplicon sequence variants (ASVs) identified as chimeras were removed, ASVs were assigned taxonomy using the Silva v132 database (Quast et al., 2012; Yilmaz et al., 2014), and nonbacterial, chloroplast, and mitochondrial reads were filtered out. Phantom reads—those from taxa present in the RNA sample but not its DNA sample counterparts—were removed from the data set (Sorensen & Shade, 2020). We used the full data set to describe the general community, but then focused our analysis on the most abundant, representative ASVs in the data set by keeping ASVs representing at least 1% of the total reads in any sample (referred to as “abundant” members of the community). For a subset of analyses below, we rarefied the data set containing the abundant ASVs to the lowest read depth (17,303 reads/sample) after inspecting rarefaction curves.

### 2.2 Statistics

After describing the living portion of ocular bacterial communities in house finches, the purpose of our statistical analyses was two‐fold. First, we aimed to assess how well microbiome data from DNA represent the living portion of ocular bacterial communities. Second, we wanted to assess our ability to detect whether taxa were active or inactive before and after antibiotic treatment, using previously described RNA:DNA ratio methods. Due to our small sample sizes, all analyses were conducted with Kruskal–Wallis tests unless otherwise specified. P‐values were adjusted with the Benjamini–Hochberg method (Benjamini & Hochberg, 1995).

With the full data set and the data set of abundant community members, we calculated the proportion of living ASVs by dividing the ASV richness in the RNA by the ASV richness in the DNA for each sample, comparing these values among treatments (pretreatment, control, antibiotic).

With the abundant community members, we then calculated alpha (with rarefied reads—richness, Shannon's entropy, Pielou's evenness) and beta diversities (Bray–Curtis, Jaccard) in QIIME2. Bray–Curtis distances were calculated from a proportional data set as suggested by McKnight et al. (2019). Alpha diversities were compared between DNA and RNA samples, treatment (pretreatment, control, antibiotic), and a proxy interaction variable that incorporated both nucleic acid type and treatment. We used the vegan package (Oksanen et al., 2020) to compare beta diversity metrics using PERMANOVAs (adonis) between DNA and RNA samples, treatment, and their interaction, and we verified that beta dispersion did not differ between nucleic acid or treatment for each distance metric. Pairwise posthoc PERMANOVAs were conducted with the RVAideMemoire package (Hervé, 2018). We visualized beta diversity with non‐metric multidimensional scaling plots. We further assessed the correlation between DNA‐ and RNA‐based samples by comparing beta‐diversity distance matrices with Mantel tests.

The remaining analyses focused on changes in communities between the two sampling times (before and after antibiotic administration) in birds given ocular antibiotic or control treatment. First, we calculated the change in the proportion of living ASVs by subtracting values before antibiotic treatment (or control) from the values after treatment per bird and compared those changes between treatments. We similarly calculated changes in richness and each alpha diversity metric within each nucleic acid sample type (DNA or RNA), and extracted pairwise beta diversity distances between pre‐ and posttreatment samples from the same bird. Next, we calculated the change in relative abundance (proportion of the reads) of each ASV between the two sampling times within each bird. We analyzed these changes in diversity and differential changes in relative abundance between DNA and RNA samples, treatments, and the interaction proxy variable.

Finally, we used the rarefied data to detect shifts between active and inactive states after antibiotic or control treatment. For the abundant ASVs, we calculated RNA:DNA ratios (Aanderud et al., 2016; Bowsher et al., 2019; Sorensen & Shade, 2020). Because rarefying creates apparent phantom reads, we added 1 to any value of ASV reads in DNA associated with >0 value in its paired RNA sample. Three RNA:DNA threshold cut‐offs were used to delineate active from inactive (RNA:DNA > 0.5, 1, or 2; Aanderud et al., 2016; Bowsher et al., 2019). Here, a cut‐off of 0.5 assumes that only half of an ASV population is generally active, but this cut‐off alternatively accounts for increased 16S rRNA gene copies per cell and extracellular, relic DNA from dead cells. The cut‐off of 1 allows for less relic DNA and requires most or all of the bacteria in an ASV to be living and translating the 16S rRNA gene. Likewise, the cut‐off of 2 assumes more rRNA than gene copies per bacterial cell. For ASVs that experienced changes in activity, we coded shifts to inactivity as −1, shifts to activity as 1, and no shift as 0, using Mantel tests to compare Euclidean distance matrices of activity changes between the threshold cut‐offs. We then used these data to qualitatively compare changes between activity states in control and antibiotic‐treated birds.

## 3 RESULTS AND DISCUSSION

**Figure A1:**
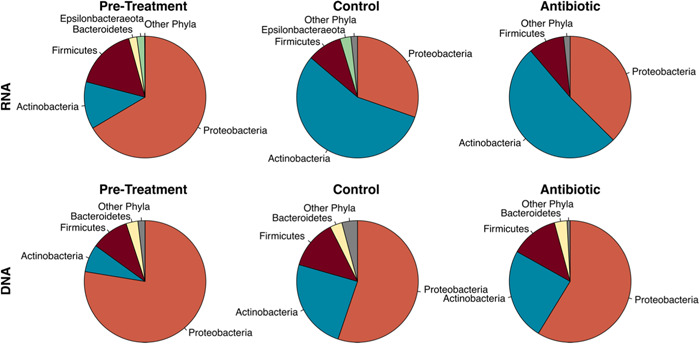
Phyla in ocular communities from RNA and DNA samples from house finches. Data represent all amplicon sequence variants (ASVs). Pies are split by treatment (pretreatment, control, antibiotics). Segments are average relative abundance. Included phyla account for at least 2% of the reads in each sample group. Names provided are based on the SILVA v132 database. See Oren and Garrity (2021) for current names.

**Figure A2:**
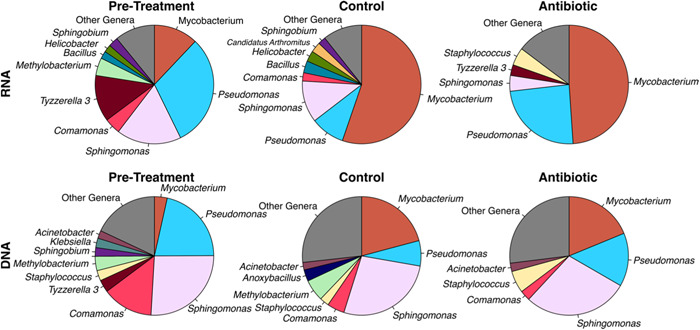
Genera in ocular communities from RNA and DNA samples from house finches. Data represent all amplicon sequence variants (ASVs). Pies are split by treatment (pretreatment, control, antibiotics). Segments are average relative abundance. Included genera account for at least 2% of the reads in each sample group.

We used bacteriostatic antibiotic treatment on house finch conjunctiva to experimentally assess the use of DNA and RNA amplicon sequencing data to describe the total and living portion of bacterial communities and shifts in activity of community members after experimental antibiotic treatment. The DNA‐based ocular bacterial communities included 802 ASVs, with 48,902 ± 20,135 reads per sample (DNA = 19,821–58,962, RNA = 28,721–83,021 reads per sample). Communities representing the living bacteria, defined here by taxa present in the RNA samples after phantom reads were removed, contained 297 ASVs and were mostly comprised of *Proteobacteria* and *Actinobacteria* (now *Pseudomonadota* and *Actinomycetota*, respectively; Oren & Garrity, 2021), particularly the genera *Mycobacterium*, *Pseudomonas*, and *Sphingomonas* (Figures A1 and A2).

We focused our analyses on the ASVs that represented >1% of the total reads for any community (RNA or DNA) because those taxa are more likely to be functionally important in the community. Bacteria with the highest relative abundances were represented by 56 ASVs (within 44 genera; 17,303–79,986 reads per sample), 11 of which were present in every sample. These abundant ASVs were also predominately in the genera *Mycobacterium*, *Pseudomonas*, and *Sphingomonas*, alongside additional rarer taxa.

Though there are limited data on ocular bacterial communities in wild animals, we can make some comparisons between our data and the known, living portion of communities in humans. Using culture‐based methods, Perkins et al. (1975) found a substantial number of isolates from healthy human conjunctiva corresponding with taxa commonly found on human skin, such as *Staphylococcus* and *Propionibacterium* species. Through amplicon sequencing, others have verified that these taxa make up a large proportion of conjunctival bacteria (Wen et al., 2017). In our healthy birds, we also found *Staphylococcus* ASVs in the RNA portion of all of our samples, as well as *Corynebacterium* ASVs (also present in healthy humans; Ozkan et al., 2017; Perkins et al., 1975), though they did not constitute a large portion of the communities. *Pseudomonas*, a large component of our samples, is generally discussed as a pathogen in ocular communities, though as an opportunistic pathogen, *Pseudomonas* may commonly be found in healthy communities alongside other commensals that balance out its abundance (Ozkan & Willcox, 2019; St. Leger & Caspi, 2018). A disproportionate amount of the living community, on the other hand, represented a single *Mycobacterium* ASV, which increased in relative abundance between the pre‐ and posttreatment time points. *Mycobacterium* has not been documented in healthy human conjunctiva, according to our literature search.

We have reported elsewhere (Weitzman et al., 2021) that the bacterial communities detected in this study were vastly different from those previously detected in house finch conjunctiva using DNA amplicon sequencing (Thomason, Leon, et al., 2017). Notably, *Lactococcus* reads had a high relative abundance in house finches from the same population a few years before the present experiment, though this genus was largely absent in our samples.

### 3.1 Describing communities with DNA vs RNA samples

**Figure 1:**
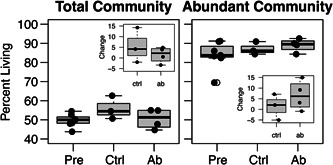
Proportion of the house finch ocular bacterial communities that were living based on RNA and DNA amplicons. The total community includes all amplicon sequence variants (ASVs) excluding phantom reads. Abundant community includes only ASVs representing at least 1% of any community. The living portion (%) of communities, and their changes across time (inset graphs), did not differ between treatments (Kruskal–Wallis: Total community, *p* ≥ 0.1 each; abundant community members, *p* ≥ 0.3 each).

**Figure 2:**
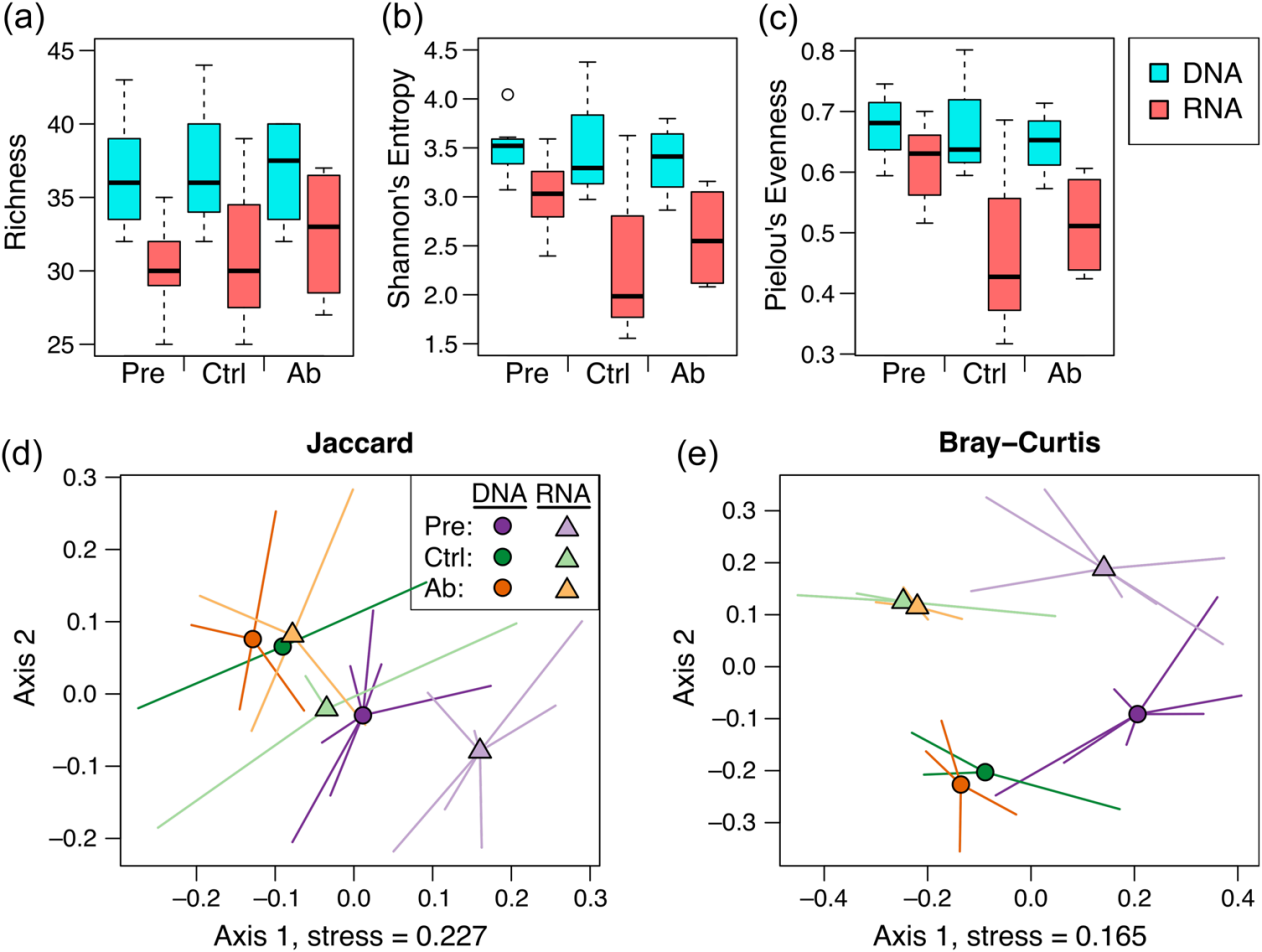
Bacterial diversity in DNA and RNA samples from house finch ocular swabs. (a–c) Alpha diversity metrics, color‐coded by nucleic acid sample type (blue = DNA, orange = RNA), with data further split by treatment (pretreatment = “Pre,” control = “Ctrl,” antibiotic = “Ab”). DNA and RNA values significantly differ (*p* < 0.05). (d, e) NMDS of beta diversity distance metrics, color‐coded by treatment. Purple = pretreatment, green = control, orange = antibiotic. Circles and darker hues = DNA, triangles and lighter hues = RNA. Diversity data are on abundant amplicon sequence variants. NMDS, non‐metric multidimensional scaling.

As discussed elsewhere (Emerson et al., 2017), using RNA to describe bacterial communities can potentially provide a clearer picture of the living bacterial community than data from DNA. Within the ocular communities described here, 44%–63% of the bacterial DNA‐based ASVs were part of the total living communities (i.e., the proportion of DNA ASVs present in the corresponding RNA sample when considering the total community before filtering for the abundant community members, Figure 1), with over 69% of the abundant community defined as living in every sample. For comparison, the average amount of extracellular DNA found in mammalian gut samples is approximately 35% (Lennon et al., 2018), and alongside extracellular DNA, over 20% of the cells in human gut communities may be inactive (Lennon & Jones, 2011). Since the RNA ASVs were subset from the DNA ASVs, we expected some alpha diversity metrics to be similar or lower in the RNA versus DNA samples. Indeed, most alpha diversity metrics measured had lower values in RNA versus DNA samples (Figure 2, Table 1), and nucleic acid type was a significant predictor of all diversity metrics assessed, indicating different community structure and composition detected by DNA‐ versus RNA‐based data.

**Table 1:** Results of alpha and beta diversity analyses across nucleic acid type (DNA vs. RNA) and treatment (three categories: before antibiotic or control treatment, after control treatment, after antibiotic treatment).

Response	Predictor	Test statistic	*R* ^2^	Adjusted *p*
Richness	Nucleic acid	8.58		**0.01**
	Treatment	0.47		1
	Interaction	9.38		0.3
Shannon's entropy	Nucleic acid	8.12		**0.02**
	Treatment	1.00		0.8
	Interaction	9.23		0.3
Pielou's evenness	Nucleic acid	7.35		**0.02**
	Treatment	1.90		0.7
	Interaction	9.64		0.3
Jaccard	Nucleic acid	5.42	0.15	**0.01**
	Treatment	3.74	0.20	**0.008**
	Interaction	0.94	0.05	1
	Mantel: DNA v RNA	0.73		**0.008**
Bray–Curtis	Nucleic acid	8.11	0.19	**0.008**
	Treatment	5.60	0.26	**0.008**
	Interaction	0.59	0.03	1
	Mantel: DNA v RNA	0.69		**0.008**

*Note*: Analyses of richness and alpha diversity were Kruskal–Wallis tests, with test statistics as *χ* ^2^. The interaction predictor variable for Kruskal–Wallis tests was a proxy variable that incorporated both nucleic acid and treatment. Beta diversity was analyzed with PERMANOVAs, for which the test statistic was *F*. Beta‐dispersion never differed in these analyses. Where treatment was significant, we only found pairwise differences between pretreatment and posttreatment samples, not between the two treatments within a timepoint. Mantel tests compared distance matrices from DNA and RNA ASV tables, with test statistic *r*. In Mantel tests, the significant result indicates a correlation between the matrices. Bold indicates significance at <0.05 cut‐off.

While DNA data are useful in indicating any bacteria that have recently encountered the ocular surface, many taxa do not seem to live or survive in the ocular community. In the unfiltered data, two families (*Deltaproteobacteria: Bdellovibrionaceae* [now *Bdellovibrionoia: Pseudobdellovibrionaceae*], *Bacilli: Thermoactinomycetaceae*) and five genera (*Actinobacteria: Nocardioides, Bacilli: Atopostipes, Deltaproteobacteria: Bdellovibrio, Gammaproteobacteria: Cupriavidus*, and *Gammaproteobacteria*: unknown *Burkholderiaceae* [now classes *Actinomycetia, Bacilli, Bdellovibrionia, Betaproteobacteria*, and *Betaproteobacteria*, respectively]) were present in most DNA samples, but were completely absent from all RNA samples, though these taxa represented a fraction of the total relative abundance (<1% each). Furthermore, the majority of taxa removed in the filtering process (i.e., relative abundance <1%; 502 of the 802 ASVs) were only present in DNA samples, revealing that the richness of rare, dead taxa in ocular communities is quite high. Although some of these may be spurious taxa (Reitmeier et al., 2021), the high proportion of dead taxa is nonetheless striking.

Using DNA to describe and compare bacterial communities is generally an accepted and useful practice (Bharti & Grimm, 2021). For example, researchers have discovered community differences between microenvironments in the same general habitat where the same transient microbes are likely present, such as on different host species in a pond (McKenzie et al., 2012). Similarly, changes or differences in communities that arise from invasions into the community (e.g., by a parasite or pathogen) can also be detected with DNA‐based methods (Jani & Briggs, 2014; Muletz‐Wolz et al., 2019; Thomason, Leon, et al., 2017). Understanding the source of differences between DNA and RNA samples is important to estimate their effects on community data (Lennon et al., 2018). If these differences stem from environmental (extracellular) DNA or transient microbes, their presence in DNA samples may be patchy and unpredictable. Alternatively, if the differences largely stem from natural cell death within the community, comparisons of interest using DNA should also largely follow differences among the living communities of interest. For example, in one study on relic DNA, Lennon et al. (2018) found that even though relic DNA can contribute anywhere from 0% to over 60% of the bacterial DNA in a mammalian fecal sample, it does not generally affect bacterial alpha or beta diversity patterns in those samples. Similarly, in our study, though there were key differences between the DNA‐ and RNA‐based data, Mantel tests of the distance matrices within each beta diversity metric suggested similar patterns are present for both data types (Table 1). This correlation between distance matrices also explains why the interaction term between nucleic acid and treatment was never a significant predictor of community diversity. Nonetheless, in other systems or cases, RNA may provide a more realistic representation of the living community, particularly when assessing functional changes or less discernable differences that may get obscured by relatively abundant dead cells.

**Figure 3:**
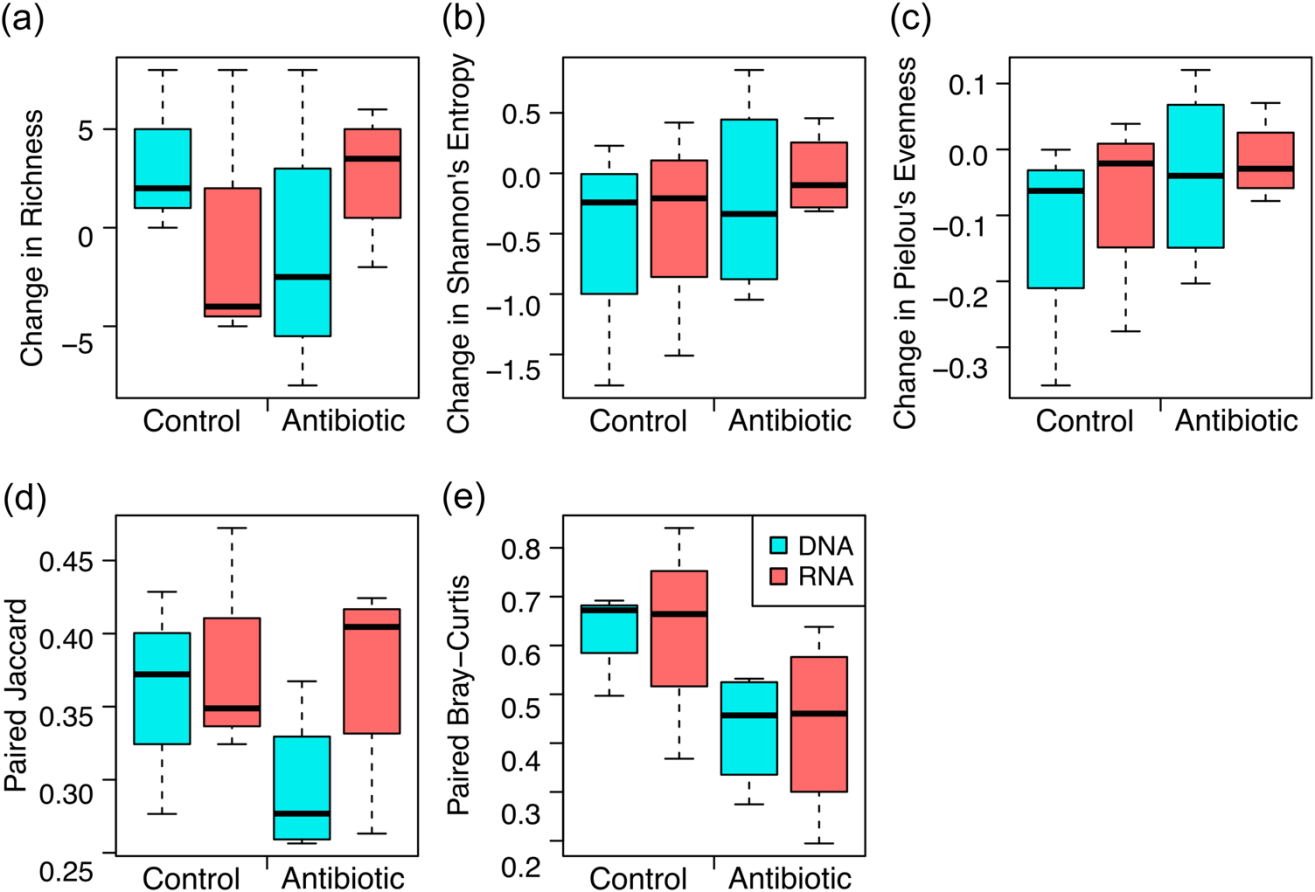
Changes in metrics of bacterial diversity within an individual over time after antibiotic or control treatment, as assessed by both DNA‐ and RNA‐based sequencing. Alpha diversity metrics include changes in (a) richness. (b) Shannon's entropy. (c) Pielou's evenness. Beta diversity metrics include paired. (d) Jaccard and (e) Bray‐Curtis dissimilarity. Data color‐coded by nucleic acid sample type (blue = DNA, orange = RNA). Changes in metrics over time were not significantly predicted by nucleic acid type, treatment, or their interaction based on Kruskal–Wallis tests, with each adjusted *p* > 0.4.

We used paired, longitudinal samples for each bird between two sampling days to determine whether detectable changes between the two time points were similar in the DNA and RNA samples. Indeed, we found that DNA and RNA samples experienced similar shifts over time, with no significance of nucleic acid type on changes in diversity over time (Figure 3), despite their general differences in diversity. A similar pattern of taxa in RNA samples tracking taxa in DNA samples has also been found in other systems. Wu and Liu (2018) used both DNA and RNA amplicon sequencing to determine ecological effects shaping protist communities. Though using both methods uncovered seasonal differences in factors affecting protist community assembly, they also generally found similar community trends for both DNA‐ and RNA‐based methods.

**Figure A5:**
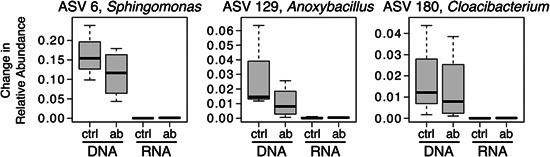
Significant changes in the relative abundance of individual amplicon sequence variants (ASVs) by nucleic acid type.

Even though RNA and DNA can detect similar patterns across space or time, changes in the relative abundance of some bacterial taxa in our bird ocular samples were significantly different between the two nucleic acid sample types (Figure A5). These bacteria increased in relative abundance in the DNA portion of the sample without similar increases in RNA, though the differences between DNA and RNA were due to their complete or near absence in the RNA portion of the samples. This further suggests that RNA‐based amplicon sequencing may be more informative, depending on the question of interest. However, comparisons of relative abundance between the two data types will be inherently skewed by the presence of fewer taxa in RNA samples, and thus higher proportions represented by the remaining taxa. Consequently, the dynamics of bacteria in DNA samples, and their comparisons with RNA samples, should be interpreted with caution.

Importantly, due to the paired nature of our RNA and DNA data, we defined the living bacterial taxa based on the DNA taxa by removing phantom reads. Through this step, we and others (e.g., Sorensen & Shade, 2020) assume that DNA samples better represent the complete living and dead community. However, if we had limited our data collection to just RNA, 731 additional ASVs would have been included in the data set, though the vast majority would be too low in proportion to be included in a data set focused on the abundant taxa in the community. Studies indicate that while phantom reads may be high and are likely a result of sampling effort, their exclusion from analyses may not impact conclusions regarding community activity since they generally represent low‐abundance taxa (Bowsher et al., 2019; Klein et al., 2016).

### 3.2 Antibiotic perturbation and community stability

**Figure A6:**
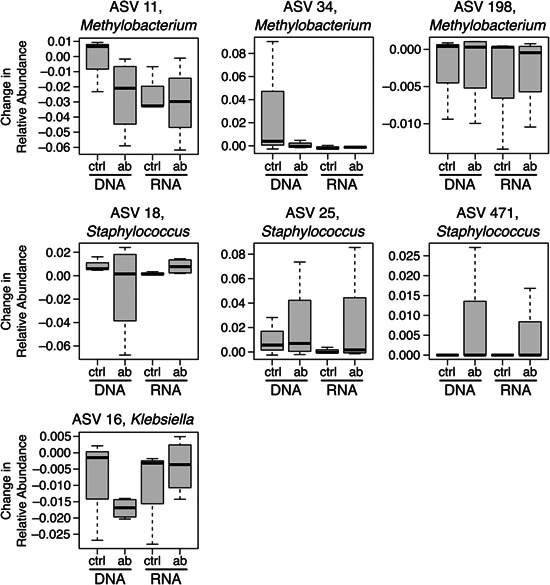
Changes in the relative abundance of amplicon sequence variants (ASVs) in genera predicted to be susceptible to cefazolin. These changes were not significantly predicted by nucleic acid type or treatment.

In addition to describing differences between DNA versus RNA communities due to factors such as dormancy or the death of transient bacteria, other experiments may specifically focus on disturbing communities to cause bacterial death (or inactivity) or survival across experimental treatments. Especially in low biomass bacterial communities, these studies may not find a strong signal of treatment differences from DNA‐based samples. In our ocular samples, we were unable to detect changes in bacterial communities after antibiotic treatment using RNA amplicon data, contrary to our prediction that there would be greater changes in RNA than DNA samples after treatment with a bacteriostatic antibiotic. We never found a significant interaction between treatment and nucleic acid type, and while the treatment itself was significant in beta diversity metrics, pairwise differences were only present between pre‐ and posttreatment groups (Table 1, Figure 2). Thus, antibiotic and control communities never differed in diversity or proportion of living ASVs (Figure 1; on a per swab basis, the number of ASVs in RNA divided by the number of ASVs in DNA). Similarly, we expected to find changes in the RNA‐based relative abundances of taxa previously found to be susceptible to the antibiotic cefazolin, such as *Methylobacterium, Staphylococcus*, and *Klebsiella* (Brown et al., 1992; Reller et al., 1973) but found no significant changes in ASVs representing these taxa (Figure A6).

The antibiotic we used in this study was chosen to disrupt the composition of the biologically active members of communities, without necessarily killing a large portion of the bacteria. The antibiotic had previously affected ocular communities enough to affect disease outcomes in the presence of an invading pathogen (Thomason, Mullen, et al., 2017). Though we were able to impact the viable community members with this antibiotic (confirmed with bacterial culturing; Weitzman et al., 2021), those changes in culture viability did not translate to changes in RNA amplicons. Importantly, this result may also be a consequence of the method used to detect bacterial differences. Amplicon sequencing methods standardize the amount of DNA (or cDNA) in the sequencing pool across samples and therefore do not address changes in absolute abundance. If the antibiotics reduced bacteria fairly evenly across taxa, amplicon sequencing would not detect changes across time, despite culturing indicating less active bacteria after antibiotic treatment. The culture‐based and culture‐independent results are thus not inherently inconsistent with each other.

We hypothesize that our use of a bacteriostatic, as opposed to bactericidal, antibiotic impacted our ability to detect significant shifts in the bacterial communities with amplicon sequencing. As reviewed elsewhere, inactive or dormant bacteria may still have relatively high levels of rRNA, with variable levels of rRNA per cell among bacterial taxa (Blazewicz et al., 2013). Studies on bacterial dormancy generally focus on soil bacteria, where nutritional availability can be measured and manipulated. In the ocular system, we know little about nutritional availability and the ecological dynamics of bacterial communities. Consequently, predicting the relative abundance of metabolically active ocular bacteria from rRNA sequencing is currently challenging.

Community changes from the pre‐ to posttreatment timepoints indicate stark changes in the ocular microbiome across time but are not necessarily attributed to antibiotic perturbation (Figure 3), suggesting that the antibiotic did not dramatically affect community composition. Changes in the proportion of the taxa in communities that were living also did not differ by antibiotic treatment (Figure 1). Similarly, changes in community diversity over time never differed based on the interaction between antibiotic treatment and nucleic acid type, though low sample sizes affected the power to detect such differences. Overall, these changes through time in both the control and antibiotic‐treated birds suggest that other factors, such as changes in housing that occurred just before the initial sampling, may have been more important drivers of ocular bacterial community composition.

### 3.3 Using RNA:DNA ratios to detect changes in bacterial activity

One method by which others have attempted to detect bacterial activity is with RNA:DNA ratios (e.g., Aanderud et al., 2016; Bowsher et al., 2019; Sorensen & Shade, 2020). Considering the known decline in bacterial growth of the ocular communities after bacteriostatic antibiotic administration (Weitzman et al., 2021), we used our amplicon sequencing to test the RNA:DNA ratio method of detecting shifts in bacterial activity in this system, with the prediction of finding a shift for many bacteria from an active to inactive state. We calculated RNA:DNA ratios of abundant ASVs in a rarefied data set, further using these data to compare three thresholds used to define ASVs as “active.” Here, active ASVs are those with RNA:DNA ratios greater than or equal to the thresholds of 0.5, 1, or 2, while inactive ASVs are defined as those with ratios below the threshold values. From these ratios, we expected to find changes to an inactive state in taxa purportedly susceptible to the antibiotic (e.g., *Methylobacterium, Staphylococcus*, and *Klebsiella*; Brown et al., 1992; Reller et al., 1973).

**Figure 4:**
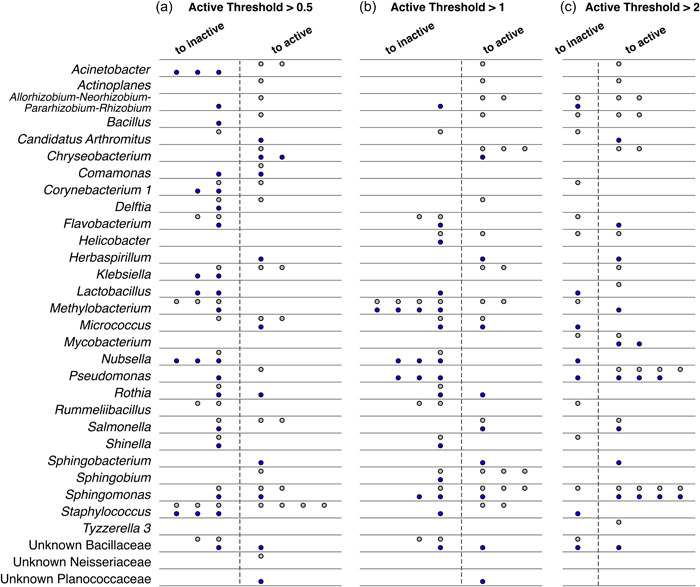
Amplicon sequence variants (ASVs) that switched status between active and inactive after ocular treatment (antibiotic or control) in captive house finches. Inactive and active are defined based on RNA:DNA ratios for an ASV. (a) RNA:DNA ratio cut‐off = 0.5. (b) RNA:DNA ratio cut‐off = 1. (c) RNA:DNA ratio cut‐off = 2. Each point represents an ASV in an individual bird that switched from active to inactive or vice versa. ASVs are grouped by genus or lowest level of taxonomy available. Gray fill (top row per genus) = antibiotic treatment (*n* = 3 birds). Blue fill (bottom row per genus) = control treatment (*n* = 4 birds). In Mantel tests, only matrices of changes in activity from cut‐offs of 0.5 and 1 were correlated (*r* = 0.3, adjusted‐*p* = 0.01; cut‐off 0.5 vs. 2, *r* = –0.01, adjusted‐*p* = 0.8; cut‐off 1 vs. 2, *r* = 0.05, adjusted‐*p* = 0.6).

The two lower thresholds provided similar results, uncovering many switches between active and inactive states, while the higher RNA:DNA threshold of 2, conversely, detected more shifts to an active state regardless of treatment group. For example, the genus *Sphingomonas* had ASVs that switched to active and inactive states in both treatments, revealed especially by the lower two thresholds (Figure 4). *Methylobacterium* exhibited similar patterns between the two treatments, with many switches to an inactive state under the low threshold cutoffs, but fewer changes under the higher ratio threshold. Similar changes between treatments complicate whether we can attribute shifts to the antibiotic or other factors experienced by the birds. *Staphylococcus* and *Klebsiella* ASVs exhibited increased activity after antibiotic treatment when using low active threshold cut‐offs, though some *Staphylococcus* ASVs did change to an inactive state in the lowest threshold in both control and antibiotic‐treated birds. The higher RNA:DNA threshold of two detected many shifts to activity in *Sphingomonas* and *Pseudomonas*, regardless of treatment group, suggesting that these two genera may have filled in available niche space freed up by the antibiotic and other community changes. The stark difference in activity shifts between the high and two lower thresholds reveals the generally low RNA:DNA ratio values in these ocular community data.

While RNA:DNA ratios suggest that the antibiotic administration forced some bacteria into an inactive state, this method did not detect many shifts between states in our ocular samples due to antibiotic treatment. Of the 56 ASVs in our filtered data set for seven birds, the vast majority did not shift between activity states in either treatment. Though this method has been used by others to designate active and inactive or dormant states in soil bacteria (Bowsher et al., 2019; Sorensen & Shade, 2020), it did not detect major changes in our samples, even though culture‐based methods using a media that supports a broad range of taxa verified a reduction in viable bacteria after antibiotic administration (Weitzman et al., 2021). Importantly, RNA:DNA ratio methods will inherently include taxa that were absent at one of the two time points, which may skew results if not accounted for. Overall, RNA:DNA ratios do not appear to be a robust proxy for bacterial activity in our ocular samples.

### 3.4 Comparing sequencing methods

We used amplicon sequencing from DNA and RNA samples as a tool to describe microbial communities, but these methods have several caveats. While relic DNA can result in overestimates of community richness through DNA‐based amplicon sequencing (Lennon et al., 2018), RNA‐based amplicon sequencing can also overestimate the living community, though to a lesser extent due to faster RNA degradation (Li et al., 2017). RNA‐based amplicon sequencing can also produce less accurate representations of viability in more complex microbial communities (Wang et al., 2023). Direct comparisons of read abundances from multiple methods compound the inherent shortcomings and errors of each method. For example, relic DNA and RNA, and varying gene copies and transcription rates across bacterial taxa (Nearing et al., 2021), can directly impact RNA:DNA ratios, limiting their ability to accurately detect small changes in microbial community composition.

Several studies have directly compared the accuracy and precision of RNA‐based amplicon sequencing and other methods to find reliable sequencing‐based options for studying microbial communities and overcoming the shortcomings of DNA‐based amplicon sequencing. Propidium‐monoazide (PMA) binds to extracellular DNA and DNA in dead cells, preventing DNA amplification by PCR (Nocker et al., 2007). After PMA treatment, DNA in living cells can be extracted and sequenced as usual. Still, PMA‐based techniques also overestimate living communities (Li et al., 2017). While PMA‐based and RNA‐based amplicon sequencing both perform better in characterizing simple, versus more complex, communities (Wang et al., 2021; 2023), RNA‐based sequencing provides more accurate representations of living communities than PMA‐based approaches (Li et al., 2017). Treating samples with PMA tends to provide results more similar to those from nontreated DNA‐based methods, or intermediary results between DNA‐ and RNA‐based methods (Li et al., 2017; Yap et al., 2022). Somewhat surprisingly, sequencing methods focused on whole bacterial genomes, including shotgun metagenomics, PMA‐treated shotgun metagenomics, and metatranscriptomics, have similar or worse accuracy and lower sensitivity in representing the living community than 16S rRNA amplicon sequencing (Yap et al., 2022). Collectively, these studies support the use of RNA‐based amplicon sequencing as a robust method for describing living microbial communities, including low‐biomass samples (Yap et al., 2022).

## 4 CONCLUSIONS

Symbiotic bacterial communities occupy virtually every surface of hosts, but our understanding of their ecology is limited by our ability to robustly characterize them. We used a bacteriostatic antibiotic to manipulate the communities of bird ocular surfaces to test the utility of DNA‐ and RNA‐based amplicon sequencing in detecting changes in these low‐abundance bacterial communities. While the bacteriostatic nature of the antibiotic used in this study did not cause detectable changes to bacterial communities as detected by amplicon sequencing, we exploited the shift to inactivity or death of bacteria following antibiotic treatment to test whether paired DNA and RNA amplicon sequencing could detect active versus inactive bacterial states. However, our results did not support the utility of using RNA:DNA ratios to detect inactive bacteria in this system.

More broadly, we explored the use of RNA‐based methods for characterizing variation in host‐associated microbiomes within and among individuals. Though we found some changes in bacterial communities between the sample days to be generally similar between RNA and DNA data, there were nevertheless dramatic differences in diversity between communities when described by different nucleotide types. Regardless, large differences or changes in communities detected from DNA, which is often the more convenient option when describing microbiomes from wild animals, may adequately represent differences in living communities. Overall, this study supports the use of DNA‐based amplicons to make broad comparisons of bacterial communities. However, the influence of DNA from dead microbial cells may cause DNA‐based methods to overrepresent some bacteria, and a nucleic acid sample type should be decided with the source of those dead cells as a consideration.

## AUTHOR CONTRIBUTIONS

**Chava L. Weitzman**: Conceptualization (equal); data curation (lead); formal analysis (lead); investigation (lead); visualization (lead); writing—original draft (lead); writing—review and editing (equal). **Dana M. Hawley**: Conceptualization (equal); funding acquisition (lead); investigation (supporting); project administration (lead); resources (equal); supervision (lead); writing—review and editing (equal). **Bahman Rostama**: Conceptualization (supporting); investigation (supporting); writing—review and editing (equal). **Meghan May**: Conceptualization (equal); funding acquisition (supporting); resources (equal); writing—review and editing (equal). **Lisa K. Belden**: Conceptualization (equal); funding acquisition (supporting); project administration (supporting); resources (equal); supervision (supporting); writing—review and editing (equal).

## CONFLICT OF INTEREST STATEMENT

None declared.

## ETHICS STATEMENT

Experimental procedures were approved by Virginia Tech's Institutional Animal Care and Use Committee.

## Figures and Tables

**Figure A3:**
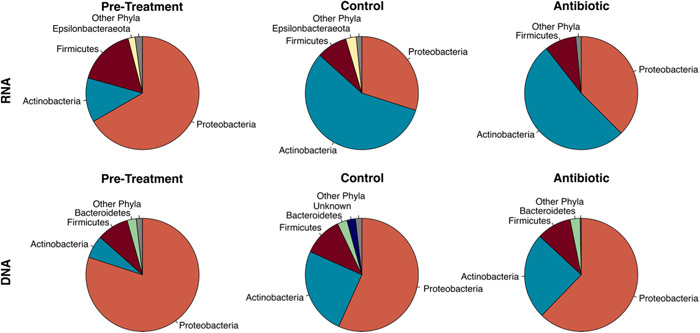
Representative phyla in ocular communities from RNA and DNA samples from house finches. Data are from abundant amplicon sequence variants (ASVs) comprising >1% of the reads from any sample. Pies are split by treatment (pretreatment, control, antibiotics). Segments are average relative abundance. Names provided are based on the SILVA v132 database. See Oren and Garrity (2021) for current names.

**Figure A4:**
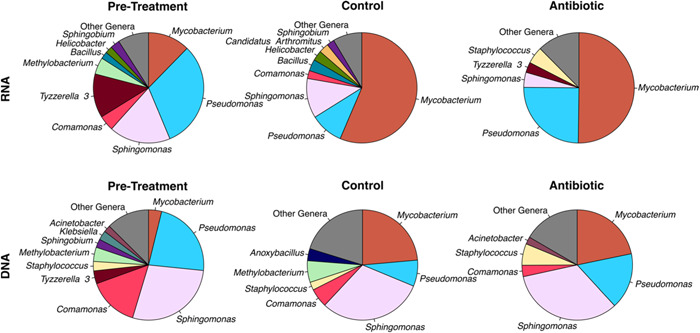
Representative genera in ocular communities from RNA and DNA samples from house finches. Data are from abundant amplicon sequence variants (ASVs) comprising >1% of the reads from any sample. Pies are split by treatment (pretreatment, control, antibiotics). Segments are average relative abundance. Included genera account for at least 2% of the reads in each sample group.

## Data Availability

Amplicon sequences are available in the NCBI database under the BioProject accession PRJNA947769: https://www.ncbi.nlm.nih.gov/bioproject/PRJNA947769.

## 1

Summary:

Figures A1, A2 provide pie charts of taxa present in the total communities, while Figures A3, A4 include taxa in abundant communities. Figure A5 contains data on significant changes in the relative abundance of ASVs between DNA and RNA data. Figure A6 provides changes in the relative abundance of ASVs predicted to be affected by the antibiotic administration.
